# The attributes of public health leadership

**DOI:** 10.1093/eurpub/ckaf019

**Published:** 2025-03-25

**Authors:** Martin McKee

**Affiliations:** European Observatory on Health Systems and Policies, London School of Hygiene Tropical Medicine, London, United Kingdom

## Abstract

The COVID-19 pandemic highlighted the importance of public health leadership. Yet, while there were many inspirational examples, too often, it was lacking. This personal perspective reflects on the experience of the pandemic, drawing on previous reflections on the role of public health professionals. It identifies eight key attributes that a public health leader should have. These are an ability to take the initiative, a sense of curiosity, a broad perspective on health threats, a recognition that there are those who are opposed to health, a willingness to speak truth to power, confidence, and the ability to engage with leaders in other sectors, a commitment to values, and an ability to communicate. In each case, it asks whether these attributes were apparent during the pandemic and why they might be needed in the future.

## The need for leadership

For decades, public health leaders have warned about the risk of a future pandemic [1]. It was a matter of when, not if, one of the many emerging and re-emerging pathogens would develop the pandemic potential. In January 2020, one did. A novel coronavirus jumped species in China and, within weeks, spread worldwide. Yet, despite the efforts of the public health community to persuade governments of the importance of anticipating such an event, the world was unprepared. Much has been written about why countries struggled to respond promptly and effectively. Some lacked technical capacity, such as a network of laboratories and a well-trained public health workforce. But even those that, at least on paper, were well equipped often performed much less well than had been expected. Responsibility for this lies mainly with the governments responsible for protecting their populations. It was they who either failed to invest in preparedness for the pandemic or whose decisions, once it arose, either failed to help or made things worse. However, the public health community must itself take some responsibility. At a time when the communities they serve looked to public health professionals for leadership, it was often lacking, even if there were some notable exceptions. In this paper, I revisit a series of attributes that, a decade ago, I proposed should underpin public health training [2] and ask whether they can help us understand what happened during the pandemic and what needs to change to avoid making the same mistakes again. It is a personal view, and, no doubt, some will disagree. However, it does draw on an extensive literature on models of scientific advice that I, with colleagues on the UK group Independent SAGE, drew on during the pandemic [3, 4] as well as my experience as a member of the Pan-European Commission on Health and Sustainable Development [5]. In particular, some will argue that the public health professional should “stay in their lane” and limit themselves to a narrow and strictly factual evaluation of the scientific evidence devoid of any consideration of what it means for policy. This is a debate that must take place, so this paper should be seen as a contribution to it.

## Initiative

The first attribute of any leader, including those in public health, is a willingness to take the initiative, acting in ways that are proactive rather than reactive and, when required, at speed. This is especially important when faced with an outbreak of infectious disease that is growing exponentially. The importance of rapid action can be seen in models of the early phase of the pandemic, showing that acting even one week earlier in the UK might have saved about half of the lives lost in the first wave of COVID-19 [6]. The corollary is that one must be prepared to be wrong. Making decisions in the face of uncertainty is not easy. Consequently, the easy choice is to delay making one. When to decide, just like what to decide, is a matter of judgment, considering the knowledge that already exists, the time and effort that will be needed to obtain more, and the urgency of the situation. However, it will be easier if the decision is explained, including why different alternatives were rejected and any uncertainty that was considered. When the pandemic began, public health leaders in those places that experienced the earliest cases of COVID-19, such as northern Italy, were in an unenviable position. However, there was much less excuse for those elsewhere who could see what was happening in Italian hospitals but failed to advise politicians to take the measures necessary to interrupt viral transmission. Again, this principle is not unique to infectious diseases. There are many other issues where there is considerable reluctance to act promptly, leaving things until it is too late, with climate change being the most obvious.

## Curiosity

The second attribute of a successful public health leader is curiosity. When faced with uncertainty, such as that created by the emergence of a new virus, it is natural to make assumptions based on past experience. However, as historians remind us, situations change, and there is a danger that, like generals who plan to fight using the by-now obsolete tactics that won the last war, our approach has failed to adapt to changing circumstances. All knowledge is contingent. In other words, it is only right until it is found to be wrong. There were many assumptions made at the beginning of the pandemic that would subsequently be found to be wrong. Yet their influence on policy persisted long after they had been disproven. One example was the widespread view in some quarters that those infected with SARS-CoV-2 words have long-term protection against subsequent infection. This led some to advocate isolating those most vulnerable while allowing the virus to spread through the population, thereby generating the illusory goal of herd immunity [7]. Another was the view that airborne transmission was not important, leading to advice to maintain a short distance from others and to invest resources in surface cleaning [8]. In both cases, there was good reason to question these views from the outset, particularly when those promoting them spoke to experts from other disciplines. However, even as the evidence to the contrary was accumulating, those promoting these views showed little, if any, curiosity as to whether they might be wrong. The reasons why they behaved as they did go beyond what can be covered in this paper but include a range of cognitive biases, a narrow disciplinary focus (including an obsessive focus on one method, such as the randomized controlled trial, even when others are more appropriate) [9] or a lack of respect for those with other forms of expertise. In some cases, the reason may also lie in adherence to a libertarian ideology or support for vested interests, represented by those whose income would be threatened by measures to reduce airborne exposure, particularly restrictions on indoor mixing or requirements to provide ventilation. Here, as so often the case when public health arguments are struggling to be heard, one should recall the words of the American writer Upton Sinclair: “It is difficult to get a man to understand something when his salary depends on his not understanding it” [10]. A lack of curiosity is equally apparent in many other areas of public health. Too often, when advising what might work, public health experts fail to ask the equally important question of what works in what circumstances, drawing on the concept of Realist Evaluation [11] This demands that they should understand the context in which it is to be applied. How does their proposal align with the prevailing values? Does it depend on trust in authorities? Does it require a well-functioning bureaucracy? How will it be received by different groups within society, especially those that are already marginalized? Yet, while the curiosity that leads one to seek out different insights and disciplinary perspectives is important, it is insufficient. Public health professionals should also be curious about the implications of the scientific advice they are giving, particularly its feasibility. This does not mean that they should compromise on the evidence. Instead, leadership involves thinking about how one’s advice should be adapted to context and framed in ways that make it most likely that it will be accepted and implemented.

## Broad view

The third is the ability to take a broad view of health threats. SARS-CoV-2 was not just a risk to those infected by it. Overwhelmed health facilities were unable to treat those with other conditions. The measures necessary to interrupt transmission, particularly lockdowns, led to major disruption of everyday life. This, in turn, meant that children were missing education, people could not work and thus earn money, and those dependent on essential services could not obtain them. An effective public health response should have addressed these issues, which were already apparent from the start of the pandemic [12]. Yet, too often, the different health threats were portrayed as opposing each other. Thus, those who were most concerned about children missing education argued strongly against closures of schools [13], even though these were important settings for disease spread. Similarly, overly strict restrictions on movement, where people were prevented from being outside homes in the fresh air, where the risk of spread was minimal, posed a threat to mental health. Leadership involves the ability to look at the whole picture, never losing sight of the importance of interrupting transmission of the virus to calling for measures to protect those who they would harm. However, a public health perspective is more than this. Above all, it involves understanding the “causes of the causes.” It demands that we ask not just whether someone is exposed to a risk factor but why? Who holds the power that determines whether they are exposed? Is it a company that has avoided regulatory controls on its products? Is it a politician who has failed to act? Is it a system that is fundamentally corrupt? The public health leader must be able to answer these questions.

## Recognize opposition

The fourth is an understanding that not everyone is committed to policies that protect and promote health. As noted previously, the protections imposed during the early stages of the pandemic were necessary but had consequences. Although many governments put in place measures to protect businesses, many, especially those in the hospitality industry, suffered. In some cases, they and their political supporters engaged in highly misleading campaigns to end these protections. At the same time, some individuals exploited the pandemic to profiteer, using their political connections to obtain vast sums of money for personal protective equipment, tests, and other items, much of which was unusable [14]. The funds involved were thus diverted from other ways of alleviating the worst effects of the pandemic. There are many individuals and organizations that profit from the sale of commodities that threaten health and well-being. The best understood are those that comprise the tobacco industry, thanks to the availability of millions of internal documents released following court orders in the USA. This has revealed how they have used a wide range of tactics to block or delay policies that threaten their interests. These include capturing the narrative [15], undermining science [16], and a range of activities that fall within the phenomenon of denialism [17]. However, they are not alone, and it is clear that many other industries employ the same methods [15]. They include the manufacturers of alcohol, firearms, junk food, and petrochemicals. Another, which has so far received less attention, is the gambling industry. Put bluntly, today’s large gambling companies are state-authorized systems for transferring vast sums of money, in large part from the poor and desperate to their fabulously wealthy owners [18] Unfortunately, there is a considerable degree of naivety about these companies in parts of the public health community, with a failure to understand how they operate. Thus, some public health professionals are willing to collaborate with these industries by participating in organizations they fund, portraying themselves as fixing the problems their products cause. These organizations invariably take a downstream approach, prioritizing measures that are largely ineffective or even counterproductive, such as education about harms, while rejecting those measures that are known to work, such as increased taxes, reducing availability, and bans on marketing, but which would damage their funders’ interests [19]. It is, however, necessary to accept that the private sector is an essential partner in any public health response, exemplified by the rapid development of vaccines during the pandemic. Ideally, the interests of the industry and the public will be the same, or at least overlap substantially. But sometimes, they are irreconcilable, and the actions of some industries contributed to conditions that exacerbated the effects of the pandemic [20]. A public health leader must be well versed in the growing literature on the commercial and political determinants of health [21], including the innovative methods developed to understand how some industries seek to subvert public health [22].

## Speak truth to power

The fifth is the imperative to speak truth to power. This is difficult for many public health professionals employed within the government, who are consequently constrained in what they can say publicly, and which may become even more so given the outcome of the 2024 US Presidential election [23]. There is, therefore, a duty on those who are not so constrained, for example, in academia, to speak out when necessary (assuming, of course, that what they are saying is based on the best available evidence). The pandemic saw many examples of governments acting in ways that threatened health, such as removing protections too early or failing to support those affected by restrictions. In response, some will argue that politicians have popular mandate for what they do as they have been elected (for now, we must assume that the electoral system is one that reasonably represents the population, and the politician believes they are acting in the interests of the population and not other vested interests, such as party donors). Politicians should welcome a situation where experts of all sorts, including the public health community, engage through the media and in association with civil society organizations to question their policy ideas. That way, the final policies are more likely to work. Inevitably, some politicians will argue that they must balance health with other considerations. Politics is about choices, and ultimately, the politicians must decide. However, it is entirely reasonable to ask them to explain how they reach their decisions and what other factors they considered. When a policy will damage health, what benefit justifies it, and to whom does that benefit flow? When a public health leader speaks truth to power, they will often focus on the evidence, seeking to make their case based on the science. However, the public health leader should not hesitate to call out those situations when politicians are acting against the public interest, for example, when prioritizing their financial supporters' interests.

## Be confident

Sixth, the public health leader must have the confidence and the ability to engage with all who can make things happen. They should not see a meeting with a health minister as the pinnacle of ambition. First, while there are notable exceptions, many occupying that post see it as a stepping stone to higher office and will prioritize their ambitions over the population's health. Second, health ministers are often among the weakest in any government. It is notable, for example, that the health portfolio in the European Commission has, until 2024, always been allocated to one of the countries with the smallest economies. Instead, they need to engage with those who hold the power. This means heads of government, finance ministers, and officials in central banks. While the public health community often frames health as a moral issue, these people are more likely to be receptive to the equally strong arguments for co-benefits, where measures to improve health help to achieve policy objectives in other sectors [24]. The obvious example is how health, like education or physical or digital infrastructure, drives economic growth [25]. This argument has only become stronger since the pandemic, as some countries struggle to recover their workforces, although even now health is missing from key policy documents [26]. Others will be attracted to the argument that those communities that feel left behind, with high levels of the “deaths of despair,” are fertile grounds for populist and, frequently, racist politicians [27]. There are many examples of those in these positions promoting what is, in effect, a public health agenda, such as former Prime Ministers Mario Monti [28] and Gordon Brown [29], and central bankers such as Mark Carney [30], while the “Greening the Financial System” movement offers many lessons for public health [31]. This does, however, mean that the public health leader must be capable of engaging in these fora. They must be familiar with the contemporary political and financial discourse. An example is the use of Environmental, Social, and Governance indicators, which offer considerable scope for promoting healthy policies [30] but which also have come under attack from some quarters [32], making it necessary to be able to engage with the competing arguments. The public health leader must also have the confidence to engage with those from other sectors, an attribute that is too rarely found.

## Uphold values

Seventh, public health leadership must be based on values. It may seem obvious that health professionals would uphold human rights, but throughout history, there have been too many examples of how they have failed to. The abuses that occurred in Europe in the 1930s and 1940s are among the worst [33], but we should not forget that eugenics was practiced widely elsewhere. Too often, public health professionals have allowed the interests of the state to override the rights of the individual [34]. However, public health should be much more than not being complicit in human rights abuses. It should involve a commitment to see problems through the eyes of those affected by them and work with them to find solutions. This demands respect for different perspectives, even if sometimes they must be understood and challenged, for example, when they conflict with established scientific facts or the rights of others, as well as an appreciation of the inevitable power imbalances that characterize interactions with the disadvantaged groups who have most to benefit from public health policies. Fortunately, there is now an extensive body of literature on co-design and co-creation that can inform these processes [35], with many examples of good practice during the pandemic [36]. Therefore, the public health leader must be willing to listen and engage.

## Communicate

The original paper included seven attributes, but, given the experience of the pandemic, it is clear that an eighth was missing. This is the ability to communicate messages to a wide range of audiences and, importantly, combat mis- and disinformation [37]. Communication is now a core part of public health curricula [38]. It includes understanding one’s audience and tailoring messages in ways that make sense to them. This includes stressing that knowledge can change and correcting messages when needed [39]. Analogies are often helpful, taking account of common misconceptions. For example, the nature of exponential growth is frequently misunderstood but can be explained by a simple example. In a classic study, subjects were asked to visualize a pond with a small patch of duckweed [40]. The area doubles each day, filling the pond by day 10. When asked when the pond will be half full, most subjects answer around day 5 when the correct answer is day 9. Effective communication also requires active countering of disinformation, which was widespread during the pandemic and was created and spread for a variety of reasons, including monetization of the digital space, taking advantage of how false messages often spread faster than true ones and thus can be used as clickbait or to spread malware, conspiracy theories, or ideological causes, undermining trust in authority [41]. It is also important to understand cognitive biases and, as far as possible, craft messages in ways that anticipate their effects [42]. Finally, the public health leader can now use a wide array of communication media, including traditional interviews and articles, podcasts, and other forms of social media, preferably with an understanding of the opportunities and pitfalls [43].

## Conclusion

The pandemic that began in 2020 tested the public health community. In theory, they had the skills needed to respond, and in many cases, they did so very effectively. But with millions of deaths, many avoidable, we need to accept that we could have done much better. Even if much of the blame lies with the politicians, it is time to reflect on what we need to do to develop the leaders of the future, who will be required if we are to respond better to the many potentially existential threats that exist.

Key points

- Proactive leadership is crucial in public health crises.
- Curiosity helps challenge assumptions and adapt strategies.
- Broad perspectives ensure all health threats are considered.
- Speaking truth to power is essential for effective policies.
- Communication skills combat misinformation and improve trust.

Conflict of interest: None declared.

## Data Availability

No data in the paper that could be made available.

## Notes

**Provenance:** Commissioned for supplement organized by WHO.
